# Supplementary material for: High‐Speed Quantitative Nanomechanical Mapping by Photothermal Off‐Resonance Atomic Force Microscopy
Source: Small. 2025 Aug 21;21(40):e07640. doi: 10.1002/smll.202507640 (PMC12508714; doi:10.1002/smll.202507640)
Supplement: Supplementary file 1 — Supporting Information [file SMLL-21-e07640-s001.docx]

Supporting Information

High-speed quantitative nanomechanical mapping by photothermal off-resonance atomic force microscopy

*Hans Gunstheimer, Gotthold Fläschner, Jonathan D. Adams, Hendrik Hölscher*, Bart W Hoogenboom**


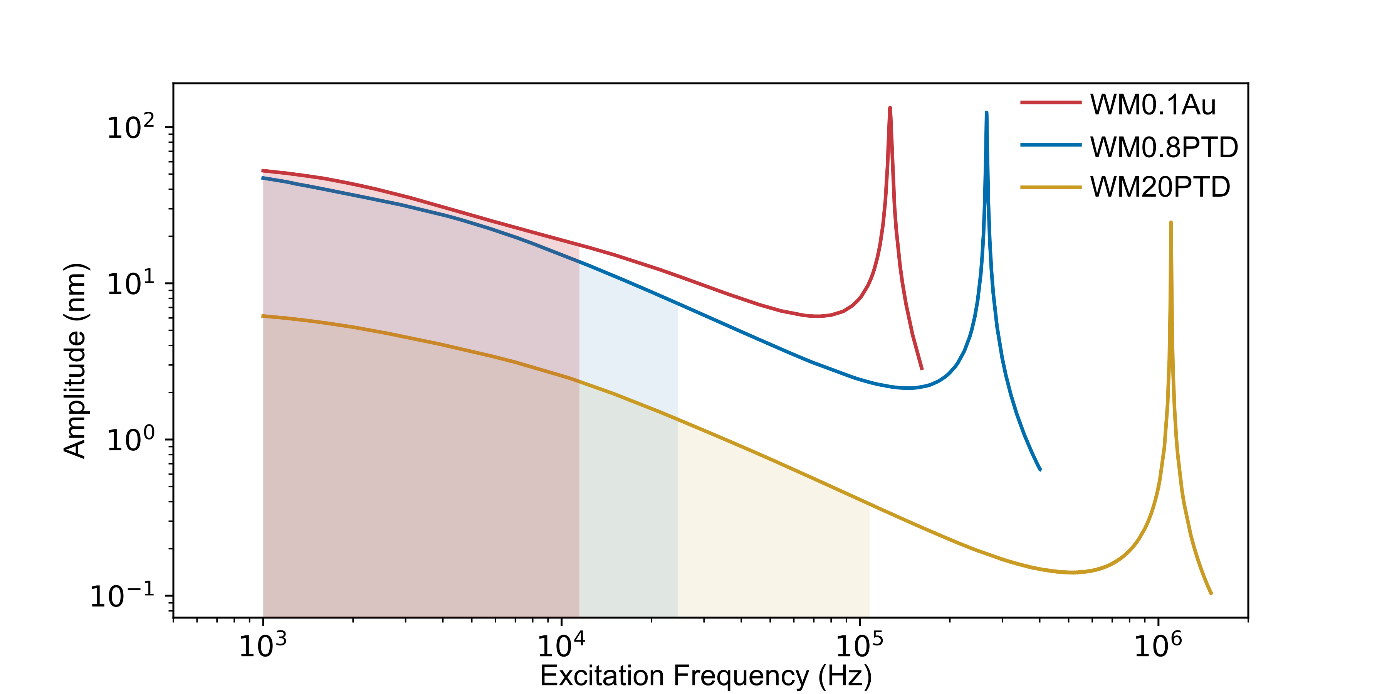
 **Supplementary Figure 1.** Frequency response of different AFM probes (WM0.1Au, WM0.8PD and WM20PTD) to photothermal excitation at 1 mW DC and 0.5 mW AC power. The amplitude excitation efficiency of a cantilever can be enhanced either by lowering the cantilever stiffness (for similar cantilever width) or optimizing the reflective coating. WM0.8PTD is softer than WM20PTD and reaches higher amplitudes for the same laser power, while the WM0.8PTD is stiffer than the WM0.1Au probe but reaches similar amplitudes thanks to optimized cantilever coating. The filled areas show the suggested frequency ranges (up to 10% of the probe resonance frequency *f*_0_) for WaveMode operation in air.


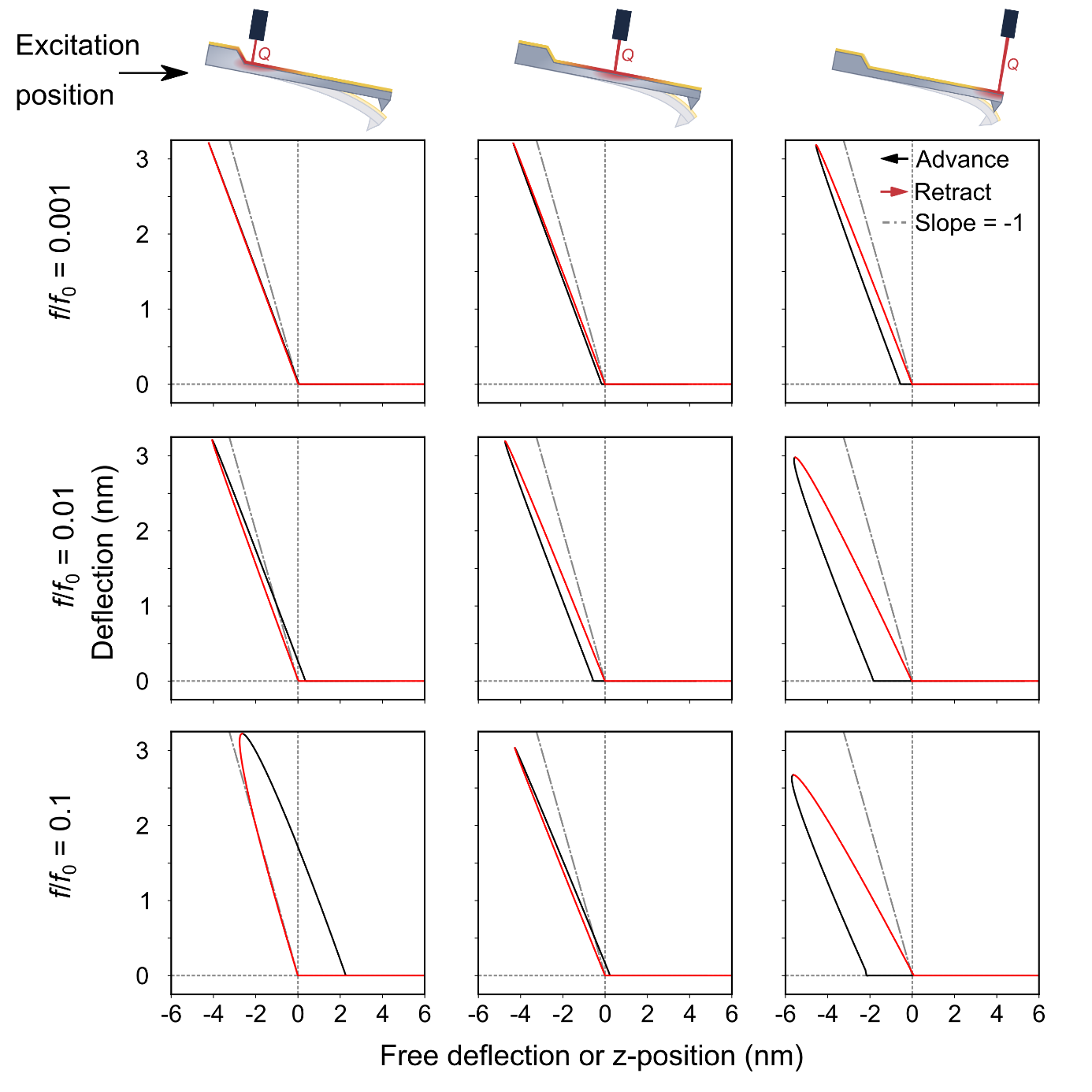


**Supplementary Figure 2.** Raw deflection-versus-position curves obtained from cantilever beam FEM-simulations with a sinusoidally disturbed temperature at the base, center and free end of the beam. A point force, with its magnitude increasing with deformation at the free end and having a force constant 1000 times higher than the beam spring constant to simulate a hard surface, was used to simulate tip-sample interaction forces. The grey, dashed line shows the expected curve for the contact region (*z* < 0) of the data, with an absolute slope of 1 nm/nm.


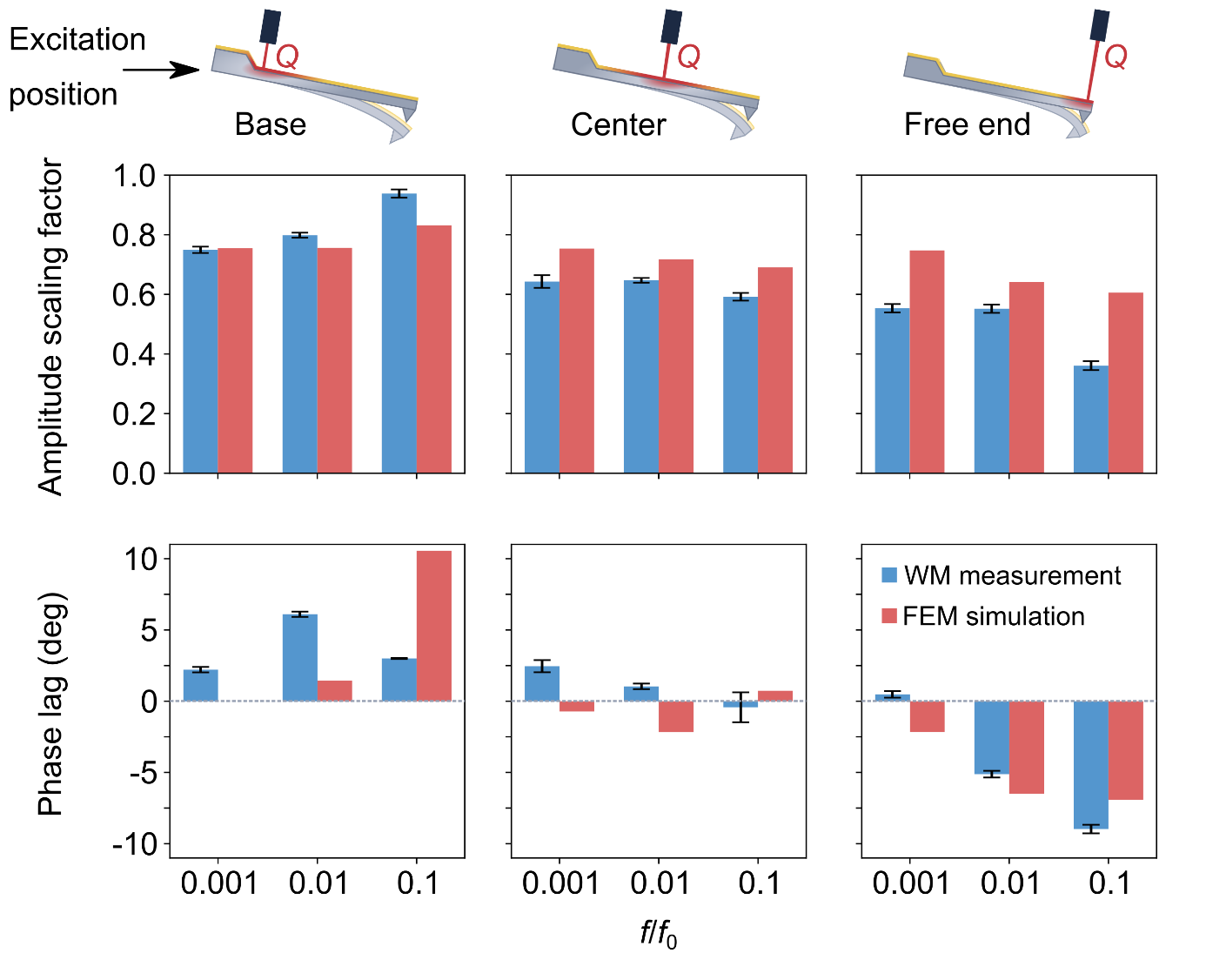
 **Supplementary Figure 3.** Amplitude and phase correction factors obtained from the measurement data against a hard surface shown in Figure 3 and cantilever beam FEM-simulation.


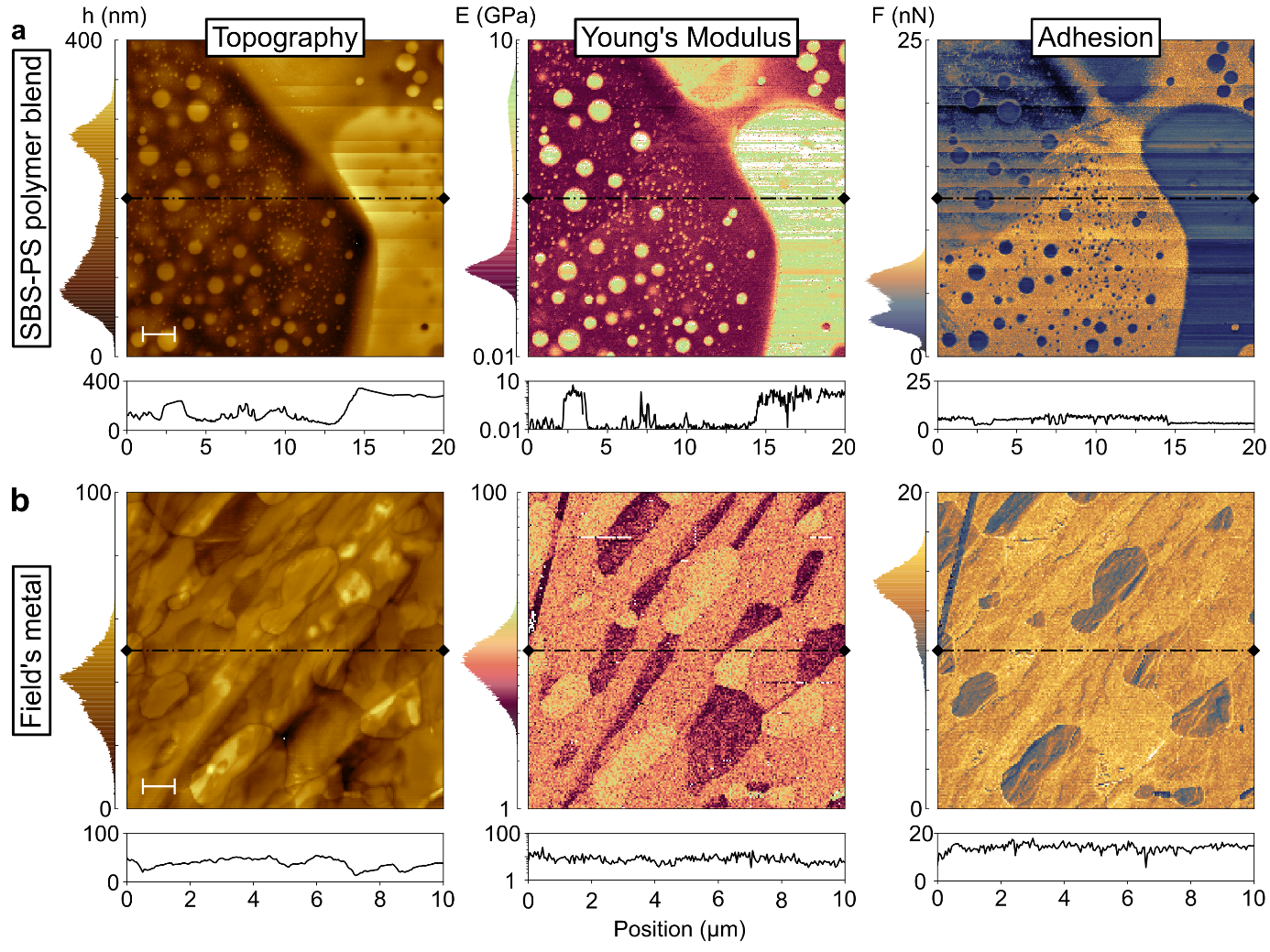


**Supplementary Figure 4.** Spectroscopy nanomechanical analysis on heterogeneous Samples.  Maps, histograms, and line profiles of (a) a polymer blend (SBS-PS), scale bar 2 µm, 420 x 420 pixels and (b) a soft metal alloy consisting of indium, bismuth and tin (Field’s metal), scale bar 1 µm, 200 x 200 pixels. Both measurements were acquired at an excitation frequency of 20 Hz (SBS-PS measurement) and 25 Hz (Field’s metal measurement), with a measurement time of (a) 147 min and (b) 27 min.


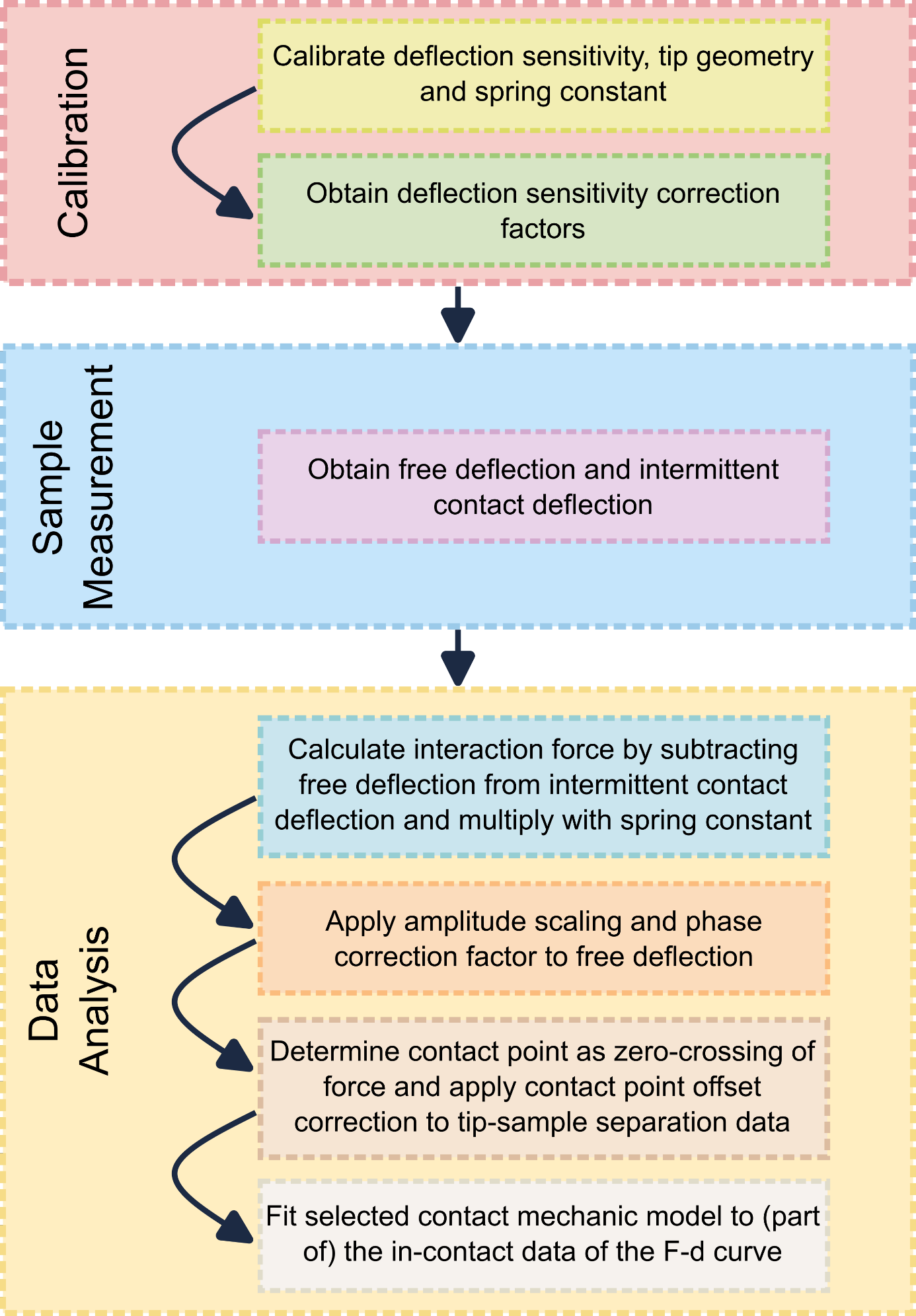


**Supplementary Figure 5.** Workflow diagram illustrating the suggested steps for calibrated nanomechanical measurements using WaveMode.

**Supplementary Table 1.** Amplitude and phase correction factors obtained from the measurement data against a hard surface shown in Figure 3 and cantilever beam FEM-simulation.

| Excitation position | Excitation frequency | Phase lag meas. (deg) | Phase lag sim. (deg) | Amplitude scaling factor meas. | Amplitude scaling factor sim |
| --- | --- | --- | --- | --- | --- |
| Base | 0.1%f0 | 2.2124 | 0 | 0.7495 | 0.7550 |
| Center | 1%f0 | 6.1002 | 1.443 | 0.7983 | 0.7558 |
| Free end | 10%f0 | 2.9970 | 10.558 | 0.9382 | 0.8311 |
| Base | 0.1%f0 | 2.4554 | -0.720 | 0.6430 | 0.7536 |
| Center | 1%f0 | 1.0404 | -2.164 | 0.6471 | 0.7175 |
| Free end | 10%f0 | -0.4230 | 0.729 | 0.5919 | 0.6907 |
| Base | 0.1%f0 | 0.4780 | -2.160 | 0.5537 | 0.7473 |
| Center | 1%f0 | -5.1228 | -6.492 | 0.5517 | 0.6411 |
| Free end | 10%f0 | -8.9730 | -6.924 | 0.3608 | 0.6057 |

**Supplementary Table 2.** Parameter of the thermo-mechanical cantilever beam FEM simulation.

| Property | Chip | Beam | Metal layer |
| --- | --- | --- | --- |
| Size (µm) | 100 x 10 | 50 x 0.5 | 50 x 0.15 |
| Mesh | Free Quad | Mapped | Mapped |
| Maximum element size (µm) | 10 | 0.5 | 0.5 |
| Minimum element size (µm) | - | 0.045 | 0.045 |
| Material | Silicon Nitride | Silicon Nitride | Gold |
| Density (kg/m3) | 3100 | 3100 | 19300 |
| Young’s modulus (GPa) | 250 | 250 | 70 |
| Poisson’s ratio | 0.23 | 0.23 | 0.44 |
| Thermal expansion coefficient (1/K) | 2.3E-6 | 2.3E-6 | 14.2E-6 |
| Heat capacity (J/(kg∙K)) | 700 | 700 | 129 |
| Thermal conductivity (W/(ms^3^K)) | 20 | 20 | 317 |

**Supplementary Video 1.** The video shows animations of the photothermal cantilever excitation at different frequencies as calculated by FEM simulations.
